# Anti-Osteoporotic Mechanisms of Polyphenols Elucidated Based on In Vivo Studies Using Ovariectomized Animals

**DOI:** 10.3390/antiox11020217

**Published:** 2022-01-23

**Authors:** Yoshimi Niwano, Hidetsugu Kohzaki, Midori Shirato, Shunichi Shishido, Keisuke Nakamura

**Affiliations:** 1 Faculty of Nursing, Shumei University, 1-1 Daigaku-cho, Yachiyo, Chiba 276-0003, Japan; kohzaki@mailg.shumei-u.ac.jp; 2 Department of Advanced Free Radical Science, Tohoku University Graduate School of Dentistry, 4-1 Seiryo-machi, Aoba-ku, Sendai 980-8575, Japan; midori.shirato.c8@tohoku.ac.jp (M.S.); shunichi.shishido.b2@tohoku.ac.jp (S.S.); keisuke.nakamura.e5@tohoku.ac.jp (K.N.)

**Keywords:** polyphenol, postmenopausal osteoporosis, ovariectomized animals, bone health

## Abstract

Polyphenols are widely known for their antioxidant activity, i.e., they have the ability to suppress oxidative stress, and this behavior is mediated by the autoxidation of their phenolic hydroxyl groups. Postmenopausal osteoporosis is a common health problem that is associated with estrogen deficiency. Since oxidative stress is thought to play a key role in the onset and progression of osteoporosis, it is expected that polyphenols can serve as a safe and suitable treatment in this regard. Therefore, in this review, we aimed to elucidate the anti-osteoporotic mechanisms of polyphenols reported by in vivo studies involving the use of ovariectomized animals. We categorized the polyphenols as resveratrol, purified polyphenols other than resveratrol, or polyphenol-rich substances or extracts. Literature data indicated that resveratrol activates sirtuin 1, and thereafter, suppresses osteoclastogenic pathways, such as the receptor activator of the nuclear factor kappa B (RANK) ligand (RANKL) pathway, and promotes osteoblastogenic pathways, such as the wingless-related MMTV integration site pathway. Further, we noted that purified polyphenols and polyphenol-rich substances or extracts exert anti-inflammatory and/or antioxidative effects, which inhibit RANKL/RANK binding via the NF-κB pathway, resulting in the suppression of osteoclastogenesis. In conclusion, antioxidative and anti-inflammatory polyphenols, including resveratrol, can be safe and effective for the treatment of postmenopausal osteoporosis based on their ability to regulate the imbalance between bone formation and resorption.

## 1. Introduction

The World Health Organization (WHO) has defined osteoporosis as a disease characterized by low bone mass and bone tissue microarchitectural deterioration that leads to enhanced bone fragility and a consequent increase in fracture risk [1]. Postmenopausal osteoporosis is a common health problem associated with estrogen deficiency [2]. Recent studies have shown that oxidative stress plays a pivotal role in the onset and progression of this disease [3], and the associated excessive generation of reactive oxygen species (ROS) has negative impacts on bone remodeling owing to osteoblast dysfunction and osteoclast activation [4,5]. Oxidative stress may enhance the expression of genes involved in inflammation, bringing about the development of postmenopausal osteoporosis [6]. Since the ROS overproduction can be associated with nuclear factor kappa B (NF-κB)-mediated inflammation [7,8], we also focused on the anti-inflammatory action of polyphenols as well as their antioxidative action. It is expected that polyphenols, with antioxidative and anti-inflammatory potential, would serve as a safe and suitable remedy for postmenopausal osteoporosis. This is consistent with epidemiological reports showing that dietary flavonoids, which are bioactive polyphenols, ameliorate bone health [9,10]. However, the fundamental mechanisms of action of these polyphenols have not yet been fully elucidated.

Aside from polyphenols’ antioxidant activity, they possess pro-oxidant potential. Typical examples of pro-oxidant activity are the antibacterial activity of catechins [11] and the cytocidal action of plant polyphenols on cancer cells [12]. Nuclear factor E2-related factor 2 (Nrf2) plays a crucial role in protecting cells, tissues, and organs on the basis of various genes encoding antioxidant proteins [13,14,15]. Upon ROS generation by polyphenols, cells could activate the Nrf2 pathway independently of polyphenols’ antioxidant activity. This idea drove us to focus on in vivo studies of polyphenols.

The purpose of this study was to comprehensively review the anti-osteoporotic effects of polyphenols in postmenopausal women. Since detailed information on this matter would be limited in human intervention studies, studies in which ovariectomized animals were used as postmenopausal osteoporosis models were screened in PubMed using the keywords “polyphenol AND ovariectomy”. The screening was further narrowed down to studies that were conducted within the last two decades (2002–2021) in which polyphenols were orally administered.

## 2. Resveratrol

Reportedly, resveratrol, a well-known sirtuin 1 (SIRT1) agonist, shows a life-span prolonging effect in various organisms [16,17,18,19,20], and it plays a beneficial role in neural, cardiovascular, and orthopedic diseases [21]. Besides such effects, it also exerts favorable effects on osteoporosis in ovariectomized rats and mice, as summarized in Table 1. Several studies have demonstrated that the oral administration of resveratrol in ovariectomized rodents mitigates estrogen deficiency-induced bone loss [22,23,24,25,26,27,28], and with respect to its anti-osteoporotic mechanisms, it has been demonstrated that resveratrol not only promotes osteoblast differentiation but also suppresses osteoclast differentiation [22,24,28]. It has been proposed that microRNAs (miRNAs/miRs) play a pivotal role in estrogen deficiency-induced osteoporosis prevention. Resveratrol suppresses the expression of miR 338 3p, which serves as a negative regulator of osteogenic differentiation in bone marrow stromal cells [29], and enhances the expression of miR-92b-3p, which inhibits osteoclast proliferation and stimulates osteoblast differentiation by suppressing the activity of the NADPH oxidase 4 (Nox4)/nuclear factor kappa B (NF-κB) signaling pathway [26]. Additionally, the administration of resveratrol to ovariectomized rats downregulates the expression of Nox enzymes, which are responsible for the production of ROS, resulting in the mitigation of estrogen deficiency-induced alveolar bone loss [27].

**Figure 1:**
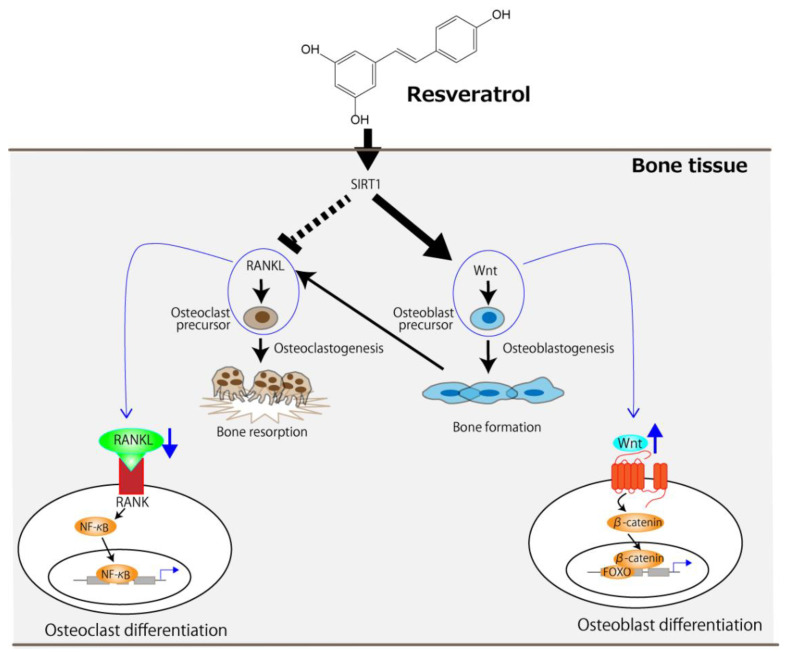
The proposed molecular-level mechanism underlying the improvement of OVX-induced osteoporosis by resveratrol centered on SIRT1. The arrows and the dotted line indicate stimulation and suppression, respectively. FOXO, forkhead box protein.

Given that resveratrol is an SIRT1 agonist, it can be concluded that SIRT1 lies at the center of its molecular mechanism. Orally administered resveratrol increases osteoblast differentiation in ovariectomized rats via the activation of SIRT1 and the subsequent suppression of NF-κB activity [22]. It has also been reported that the management of osteoporosis in resveratrol-treated ovariectomized rats could be achieved by activating two signaling pathways—namely, the SIRT1 and wingless-related MMTV integration site (Wnt) pathways [28]. The receptor activator of NF-κB ligand (RANKL), which is released by osteoblasts and is essential for osteoclast development and activation, and its receptor, RANK, signal the activation of NF-κB, resulting in accelerated osteoclast differentiation [30,31,32]. However, the activation of SIRT1 by resveratrol can suppress RANKL-induced NF-κB activity [22,28]. The Wnt signaling pathway not only promotes osteoblast proliferation but also attenuates their apoptosis [33], and this is further enhanced by SIRT1 [34,35]. Figure 1 summarizes the molecular-level mechanism of osteoporosis management, centered on SIRT1, in ovariectomized rodents using resveratrol. Resveratrol can suppress the RANKL/RANK signaling pathway in osteoclast precursors, leading to a decrease in osteoclast differentiation. It can also enhance the Wnt signaling pathway in osteoblast precursors, leading to increased osteoblast differentiation.

## 3. Purified Polyphenolic Compounds Other Than Resveratrol

The beneficial effects of purified polyphenolic compounds other than resveratrol on osteoporosis in ovariectomized rodents are summarized in Table 2. Phloridzin, an apple polyphenol, along with oleuropein, tyrosol, and hydroxytyrosol, all of which are olive oil polyphenols, elicit protective effects on bone loss in ovariectomized + inflammation-provoked rats, probably by lowering the risk of inflammation-induced osteopenia via their antioxidant activity [36,37,38]. It has also been observed that both oleuropein and hydroxytyrosol prevent bone loss in ovariectomized mice by regulating oxidative stress via their antioxidant effects [39]. Further, phytoestrogens, such as genistein and 8-prenylnaringenin, also prevent bone loss and improve bone biomechanical strength in ovariectomized rats via an agonistic action on estrogen receptors [40], and reportedly, luteolin, a plant flavonoid, significantly enhances bone mineral density (BMD) and bone mineral content (BMC), while mitigating bone strength loss in ovariectomized mice by reducing both osteoclast differentiation and function [41].

Based on these studies, we focused on the relationship between the anti-inflammatory effect of polyphenol compounds as well as their osteoporosis-alleviating effects. As a hypothesis for the relationship between estrogen deficiency, bone loss, and inflammation in osteoporosis, it has been suggested that pro-inflammatory cytokines, such as interleukin-1 (IL-1), tumor necrosis factor-alpha (TNF-α), and IL-6, are the primary mediators of accelerated bone loss during menopause [42]. The increased production of such pro-inflammatory cytokines is associated with the binding of RANKL to RANK, which results in osteoclastic bone resorption during estrogen deficiency.

## 4. Polyphenol-Rich Substances or Extracts

The beneficial effects of polyphenol-rich substances or extracts are summarized in Table 3. Tea, a popular beverage that is consumed worldwide, is prepared from the processed leaves of the plant *Camellia sinensis* using either hot or cold water. The results of some studies have indicated that green tea containing epicatechin, epicatechin-3-gallate, epigallocatechin, and epigallocatechin-3-gallate has bone-health improvement effects. It has also been reported that green tea polyphenols (GTPs) in drinking water mitigate bone loss as well as bone microarchitecture deterioration in middle-aged ovariectomized rats [43,44,45]. These beneficial effects of GTPs can be attributed to their antioxidant potential. For example, the glutathione peroxidase produced by osteoblasts protects against ROS [46], and simultaneously decreases oxidative stress damage in bone tissue. Further, safflower seed (*Carthamus tinctorius* L.) has been clinically used in Korea; specifically, defatted safflower seed powder containing polyphenols partially prevents OVX-induced bone loss in rats by stimulating osteoblast proliferation [47]. As a putative phytoestrogen, polyphenol-rich Du-Zhong (*Eucommia ulmoides* Oliv.) cortex extract prevents OVX-induced bone loss in rats [48]. Moreover, Du-Zhong, which is rich in polyphenolic compounds, such as lignans, phenolic acids, and flavonoids, is a kidney-tonifying herbal medicine used in China, and possibly, its extract stimulates osteoblast activity and inhibits osteoclast resorption via estrogen receptor β. A relatively short-term study (8 days) involving the use of dietary purified blueberry polyphenols, which are rich in anthocyanin, revealed that polyphenols enhance calcium absorption in ovariectomized rats [49]. As an interesting anti-osteoporotic mechanism, it was recently reported that arecanut (*Areca catechu* L.) seed polyphenol promotes bone formation by altering gut microbiota along with controlling inflammatory reactions [50]. The seed has also been used in osteoporosis therapy in the field of Chinese medicine. Polyphenol-rich heat-treated melon extract is also a typical example of a substance with antioxidant activity that illustrates the protective effect of polyphenols on OVX-induced bone loss [51]. Specifically, melon extract protects against the deterioration of bone strength as well as bone mineralization in ovariectomized rats, probably owing to its potent antioxidant activity.

## 5. Studies in Which Polyphenols Did Not Show Any Beneficial or Positive Effects on Bone Health in Ovariectomized Animals

Some studies have shown that polyphenols do not exert any positive effects on bone health in ovariectomized animals (Table 4). In one study, genistein, administered once daily as an oral supplement for five months, did not show any beneficial effect on the tibia in ovariectomized rats used as a model for postmenopausal bone loss [52]. In another study, the synergistic administration of quercetin, genistein, resveratrol, and vitamin D_3_ via feeding for 4 weeks failed to protect bone loss in ovariectomized aged rats [53]. Even though the feeding administration of polyphenol-rich blueberry extract showed increased calcium absorption in ovariectomized rats [49], a purified extract of blueberry polyphenols or lyophilized blueberries administered via oral gavage for 90 days failed to improve BMD, bone microarchitecture, or other mechanical properties of the bone in ovariectomized rats [54]. The results of this study emphasized that most surrogate measures of bone strength (e.g., BMD and calcium absorption) are only partially correlated with bone mechanical properties and fracture. In another study, the feeding administration of extra virgin olive oil polyphenols for 12 weeks regulated uterine estrogen response marker genes in an E2-agonistic manner but did not mitigate estrogen ablation-induced bone loss [55]. Relatively old animals were used in three of the four studies presented in Table 4; thus, waning metabolic activity due to senescence could explain the poor osteogenic effect of the polyphenols. A review on the bioavailability of phytoestrogens using rodent models [56] reported that data should be interpreted with caution because of the large interspecies variability observed in the metabolism of phytoestrogens by rodent, e.g., limited intestinal absorption as well as rapid excretion. These are limitations of animal studies involving rats and mice. Thus, when interpreting the mode of actions, care must be taken when extrapolating the results corresponding to animal models to humans.

## 6. Conclusions

**Figure 2:**
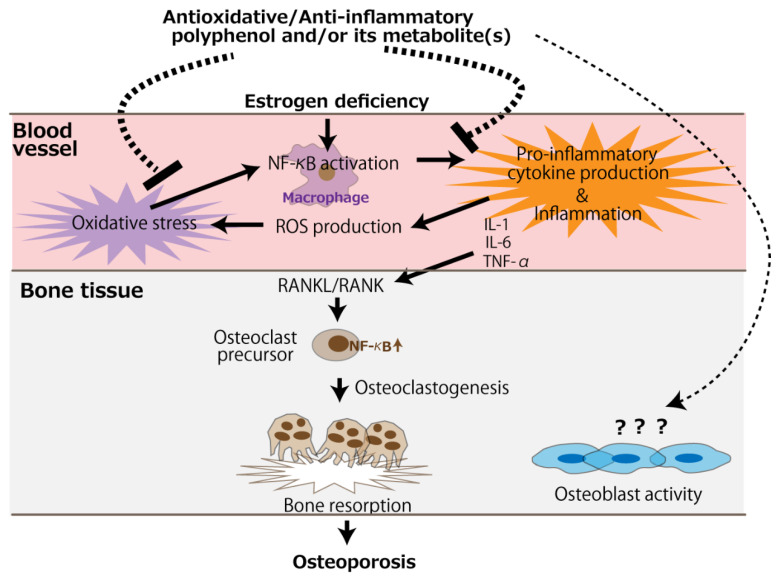
The putative mechanism of the antioxidative and anti-inflammatory effects of polyphenols for the prevention of postmenopausal osteoporosis. The arrows and dotted line indicate stimulation and suppression, respectively. ROS, reactive oxygen species.

The bone loss in ovariectomized animals is likely due to accelerated osteoclastogenic activity induced by a deficiency in estrogen, leading to the imbalance of bone turnover in which resorption outpaces formation [57]. It would be presumed that polyphenols other than resveratrol mainly counteract the accelerated osteoclast activity. Anti-osteoporotic mechanisms of antioxidative and anti-inflammatory polyphenols are schematically represented in Figure 2. Besides the direct action on osteoclasts, circulating polyphenols and/or their metabolites can suppress oxidative stress caused by the NF-κB activation of the monocyte-macrophage system under estrogen deficiency, alleviating pro-inflammatory cytokine production. Subsequently, the RANKL/RANK signaling pathway is suppressed, leading to the mitigation of osteoclastogenesis. On the contrary, evidence of an effect of polyphenols on osteoblast activity is scarce, and only two in vitro studies have reported such an effect [47,48].

To confirm the idea proposed in Figure 2, we think that information on the absorption, distribution, metabolism, and elimination (ADME) of the target polyphenols would be essential. However, instead of conducting a complete ADME study, which may be time-consuming and expensive, a simple pharmacokinetic study can also be of significance. For instance, a liver S9 fraction assay combined with a Caco-2 permeability assay and a liquid chromatography–mass spectrometry analysis can be useful [58] for the examination of the involvement of circulating metabolites in the observed action of their mother polyphenols in vivo and for the identification of their target sites of action. Future studies should also investigate the role of polyphenol pro-oxidant properties in improving bone health. If ROS produced by polyphenols, via the oxidation of phenolic hydroxyl moiety and the reduction of dissolved oxygen, have the potential to activate the Nrf2 pathway, it would provide a new perspective to the in vivo effects of polyphenols.

Finally, to further confirm polyphenols’ ability to prevent bone fragility fractures in postmenopausal females with osteoporosis, we think that more studies using aged animals focusing on bone microarchitecture and the mechanical properties of the bone—rather than surrogate measures of bone strength (e.g., BMD and calcium absorption)—are needed.

## Figures and Tables

**Table 1:** Beneficial effects of oral resveratrol on osteoporosis in ovariectomized rodents.

Reference, Year of Publication, and Animal Species with Age at OVX	Dosage	Major Improved Parameter(s)	Mode of Action	
			Overview	Molecular Level
[22] 2014 Rats aged 3 months	5, 25, and 45 mg/kg/day for 8 weeks starting at 1 week after OVX	BMD of lumbar vertebrae (L3) and femur	Increased osteoblast differentiation	Activation of SIRT1 and subsequent suppression of NF-κB activity
[23] 2014 Rats aged 3–4 months	20, 40, and 80 mg/kg/day for 12 weeks starting at 2 weeks after OVX	BMD of femur	―	―
[24] 2020 Rats aged 10–12 weeks	10, 20, and 40 mg/kg/day for 8 weeks starting at 1 week after OVX	BMD of the lumbar vertebrae (L3) and the right distal femur–tibia bone region	Promotion of osteoblast differentiation and suppression of osteoclast differentiation via autophagy regulation	―
[25] 2020 Rats aged 3 months	5, 15, and 45 mg/kg/day for 12 weeks starting at 3 days after OVX	BMD of femur	Estrogen-like activity	―
[26] 2020 Rats aged 6 months	50, 100, and 200 mg/kg/day for 12 weeks after OVX	BMD of femur	―	Suppression of cathepsin K expression and the Nox4/NF-κB signaling pathway through the elevated expression of miR-92b-3p
[27] 2020 Adult rats	10 mg/kg/day for 140 days after OVX (sacrificed 28 days after experimental periodontitis initiation)	Alveolar bone mass	―	Downregulation of NADPH oxidase levels
[28] 2021 Rats aged 3 months	80 mg/kg/day for 8 weeks starting at 8 weeks after OVX	BMC and BMD of femur	Stimulation of osteogenesis and inhibition of osteoclastogenesis	Activation of sirtuin 1 (SIRT1) and wingless-related MMTV integration site (Wnt) pathways

OVX, ovariectomy; BMD, bone mineral density; BMC, bone mineral content; ―, not clearly described. Unless otherwise stated, resveratrol was administered by oral gavage.

**Table 2:** Beneficial effects of oral purified-polyphenolic compounds other than resveratrol on osteoporosis in ovariectomized rodents.

Reference, Year of Publication, and Animal Species with Age at OVX	Dosage	Major Improved Parameter(s)	Mode of Action
Phloridzin (an apple polyphenol)			
[36] 2005 Rats aged 6 months	Oral administration with a diet supplemented with 0.25% phloridzin for 80 days after OVX. Inflammation was induced by a subcutaneous injection of magnesium silicate 3 weeks before necropsy.	BMD of femur	Improving inflammatory marker levels and decreasing bone resorption
Oleuropein (an olive oil polyphenol)			
[37] 2006 Rats aged 6 months	2.5, 5, 10, and 15 mg/kg/day for 100 days after OVX	BMD of femur	Reducing inflammatory state
Tyrosol and hydroxytyrosol (olive oil polyphenols)			
[38] 2008 Rats aged 6 months	Oral administration with a diet supplemented with either 0.017% tyrosol or 0.017% hydroxytyrosol for 84 days after OVX; Three weeks before the end of the investigation (d 63), inflammation was provoked by a subcutaneous injection of magnesium silicate.	BMD of femur	Possibly lowering the risk of inflammation-induced osteopenia via their antioxidant activity
Oleuropein and hydroxytyrosol (olive oil polyphenols)			
[39] 2011 Mice aged 6 weeks	10 mg/kg at 3-day intervals for 28 days after OVX	BMD of femur	Regulating oxidative stress via their antioxidant effects
Genistein (GEN) and 8-prenylnaringenin (8PN)			
[40] 2008 Rats aged 3 months	6 and 60 mg GEN/kg/day or 6.8 and 68 mg 8PN/kg/day for 3 months after OVX	BMD of tibia	Acting as phytoestrogens
Luteolin			
[41] 2011 Mice aged 9 weeks	5 and 20 mg/kg/day for 30 days starting at 1 week after OVX	BMD and BMC of femur	Reducing both osteoclast differentiation and function

OVX, ovariectomy; BMD, bone mineral density; BMC, bone mineral content. Unless otherwise stated, the polyphenols were administered by oral gavage.

**Table 3:** Beneficial effects of oral polyphenol-rich substances or extracts on osteoporosis in ovariectomized rodents.

Reference, Year of Publication, and Animal Species with Age at OVX	Dosage	Major Improved Parameter(s)	Mode of Action
Green tea (*Camellia sinensis*) polyphenols (GTPs) containing epigallocatechin-3-gallate			
[43] 2008 Rats aged 14 months	0.1% and 0.5% (*w*/*v*) GTP aqueous solution for 16 weeks after OVX	BMD of femur	An increase in antioxidant capacity and/or a decrease in oxidative stress damage
[44] 2009 Rats aged 14 months	0.1% and 0.5% (*w*/*v*) GTP aqueous solution for 16 weeks after OVX	BMD of femur; Bone microarchitecture of tibia	An increase in antioxidant capacity and/or a decrease in oxidative stress damage
[45] 2019 Rats aged 6 months	0.15%, 0.5%, 1.0%, and 1.5% (*w*/*v*) GTP aqueous solution for 3 and 6 months after OVX	Bone microarchitecture and mechanical properties of tibia, femur, and lumbar vertebrae (L3)	GTP’s antioxidative and anti-inflammatory actions
Defatted safflower (*Carthamus tinctorius* L.) seed powder containing lignans and flavones			
[47] 2002 Rats aged 12 weeks	290 g/kg diet for 4 weeks starting at 1 week after OVX	Bone mass of proximal tibia	Possibly stimulating osteoblast proliferation
Polyphenol-rich Du-Zhong (*Eucommia ulmoides* Oliv.) cortex extract containing lignans, phenolic acid, and flavonoids			
[48] 2009 Rats aged 3 months	100, 300, and 500 mg/kg/day for 16 weeks starting at 4 weeks after OVX	BMD of femur	Possibly stimulating osteoblast activity and inhibiting osteoclast resorption through Erβ
Polyphenol-rich blueberry (*Vaccinium corymbosum*) extract containing anthocyanins, phenolic acids, plavan-3-ols, and flavonols			
[49] 2020 Rats aged 5 months	75, 350, and 1000 mg total polyphenols/kg/day for 8 days starting at 12 days after OVX	Calcium absorption	―
Arecanut (*Areca catechu* L.) seed polyphenol containing proanthocyanidin b2, procyanidin b1, catechin, etc.			
[50] 2021 Rats weighing 190 ± 10 g	400 and 800 mg/kg/day for 90 days starting at 30 days after OVX	Trabecular microstructure of femur	Promoting bone formation by altering gut microbiota along with controlling inflammatory reaction
Polyphenol-rich heat-treated melon (*Cucumis melo* L.) extract			
[51] 2019 Rats aged 7 weeks	1 mL of the extract 3 times/day for 4 weeks starting at 8 weeks after OVX	Bone strengths of femur; BMC and BMD of whole body, femur, and lumbar vertebrae 4–6	Potent antioxidant activity leading to protection from the decline in bone strength, mineralization, and metabolism

OVX, ovariectomy; BMD, bone mineral density; BMC, bone mineral content; ―, not clearly described. Unless otherwise stated, substances or extracts were administered by oral gavage.

**Table 4:** Studies in which oral polyphenols and polyphenol-rich extracts did not show any beneficial effects on bone health in ovariectomized rodents.

Reference, Year of Publication, and Animal Species with Age at OVX	Dosage	Observations
Genistein		
[52] 2013 Rats aged 7, 16, and 22 months	A pellet/day (485 and 970 μg genistein/pellet) for 5 months after OVX	No significant effects on cancellous or cortical bone mass or architecture of tibia
Mixure of quercetin (QUE), genistein (GEN), resveratrol (RES), and vitamin D_3_ (VD)		
[53] 2018 Aged rats (retired breeder)	1000 mg QUE/kg diet, 500 mg GEN/kg diet, 200 mg RES/kg diet, and 2400 IU VD/kg diet for 4 weeks after OVX; 2000 mg QUE/kg diet, 1000 mg GEN/kg diet, 400 mg RES/kg diet, and 2400 IU VD/kg diet, for 4 weeks after OVX	No significant effects on BMD of whole femur or L4 or L5
Purified extract of blueberry (*Vaccinium corymbosum*) polyphenols or lyophilized blueberries		
[54] 2021 Rats aged 5 months	50, 250, or 1000 mg total polyphenols/kg/day for purified blueberry polyphenols or 50 mg total polyphenols/kg/day for lyophilized whole blueberries, for 90 days starting 1 month after OVX	Insignificant effects on BMD and bone mechanical properties
Extra virgin olive (*Olea europaea* var. Koroneiki) oil total polyphenolic fraction (TPF) containing oleocanthal, oleacein, and ligstroside as major polyphenols		
[55] 2014 Rats aged ~12 months	800 mg TPF/kg diet for 12 weeks after OVX	No significant effects on the bone loss of tibia

OVX, ovariectomy; BMD, bone mineral density. Unless otherwise stated, substances or extracts were administered by oral gavage.
